# Inhibition of Citrinin Production in *Penicillium citrinum* Cultures by Neem [*Azadirachta indica* A. Juss (Meliaceae)]

**DOI:** 10.3390/ijms9091676

**Published:** 2008-09-02

**Authors:** Simone Aparecida Gallerani Mossini, Carlos Kemmelmeier

**Affiliations:** Department of Biochemistry, State University of Maringá. PR, Brasil

**Keywords:** *Penicillium citrinum*, citrinin inhibition, *Azadirachta indica*, neem

## Abstract

The efficacy of different concentrations of aqueous neem leaf extract (3.12 to 50 mg/mL) on growth and citrinin production in three isolates of *Penicillium citrinum* was investigated under laboratory conditions. Mycotoxin production by the isolates was suppressed, depending on the concentration of the plant extract added to culture media at the time of spore inoculation. Citrinin production in fungal mycelia grown for 21 days in culture media containing 3.12 mg/mL of the aqueous extract of neem leaf was inhibited by approximately 80% in three isolates of *P. citrinum*. High-performance liquid chromatography was performed to confirm the spectrophotometric results. Vegetative growth was assessed, but neem extract failed to inhibit it. Neem leaf extract showed inhibition of toxin production without retardation in fungal mycelia growth.

## 1. Introduction

Due to the widespread presence of fungi in the environment, mycotoxins are considered unavoidable contaminants in foods and feeds. Citrinin contamination by *P. citrinum* has been reported in agricultural commodities, food, feedstuffs and it has been implicated in mycotoxic nephropaty [1]. Furthermore, citrinin is also embryotoxic, teratogenic and genotoxic [2]. Of late, there is considerable interest in preservation of grains by using natural products in order to effectively retard growth and mycotoxin production [3]. Owing to health and economic concerns, several searches were undertaken to discover compounds that would safely be used as substitutes for fungicides to inhibit fungus growth or mycotoxin production. The plant parts of a tree, A*zadirachta indica* A. Juss (Meliaceae), called neem has insecticidal activity with low toxicity to non target organisms [4–6]. One of the main features of neem extracts, particularly those from leaves, is that they do not cause retardation in fungal growth, but inhibit aflatoxin production [7]. *In vitro* investigations confirmed the inhibitory effects of neem on aflatoxin [8, 9] and patulin production [10]. Current research investigates effectiveness of water extracts of neem leaves on citrinin production and growth of isolates of *P. citrinum.*

## 2. Material and Methods

### 2.1. Microorganisms

Three citrinin-producing isolates of *Penicillium citrinum* (K1, K4 and K8), from the culture collection of the Laboratory of Chemistry and Physiology of Microorganisms (Department of Biochemistry, State University of Maringá, Pr) stored in silica, were used.

### 2.2. Preparation of neem leaf extract (NLE)

Leaves of *Azadirachta indica* A. Juss (Meliaceae) were obtained from the Agricultural Institute of the State of Paraná (IAPAR) located in Londrina, PR. Brazil. Dried leaves of *A. indica* were extracted by maceration in distilled water (100 g/L) and stirred for five hours in the dark, at room temperature. At the end of the extraction, the material was sieved through WHATMAN 1 filter paper (Whatman, UK), freeze-dried, and preserved in dark flasks. A 10% aqueous extract of the residue was prepared and used in this investigation [10].

### 2.3. Cultures conditions and extraction of citrinin

Citrinin was produced in liquid potato-dextrose medium (PD) as described by Wu *et al*. [11]. Inocula containing 10^5^ spores of each strain of *P. citrinum* were added to PD medium (3 mL) in 25 mL flasks (20×100mm) Pyrex® and incubated at 26±0.5°C for 21 days. Treatments in four replicates consisted of 10% freeze-dried aqueous NLE at concentrations 3.12, 6.25, 12.5, 25 and 50 mg/mL added to the PD, before autoclaving and inoculation. The flasks were weighed prior to the addition of medium, inocula and neem extract and post incubation. Dry weights were obtained by freeze-drying of controls and treatments [12]. The number of spores after incubation in controls and in treatment was obtained by counting on a haematocytometer. Citrinin was extracted from cultures three times with 10 ml of chloroform, treated with anhydrous sodium sulfate, filtered and evaporated to dryness [13]. Residues were dissolved in chloroform (0.1 mL).

### 2.4. Mycelium inhibition test

Effects of NLE on radial growth and colony characteristics of *P. citrinum* isolates were determined by growth on PDA medium containing NLE (12.5 mg/mL) and on an extract-free medium, according to the poisoned plate technique [14]. Media PDA and PDA-NLE were inoculated at the center of the plate [15]. Plates were incubated for 7 days at 25°C. Four replicates for each medium in two separate experiments were used. The radial growth of fungi was measured on each plate and compared, as described by Amandioha [16]. The macroscopic and microscopic morphological features of the colonies were visually compared after staining with 0.1% lactofuchsin staining [15].

### 2.5. Citrinin determination

Analyses were performed according to Betina [13]. Citrinin (Sigma®) standard was prepared in ethanol (1 mg/mL) and stored at 4°C. Extracts (dissolved in chloroform) and standard, underwent TLC on 20 × 20 cm Aluminium plates (Silica gel 60 – G. Merck®), with toluene-ethyl acetate-formic acid (6:4:0.5, v/v). After development the plates were exposed to 365 nm ultraviolet light (UV). Citrinin appears as a fluorescent yellow spot. The phenolic group in citrinin, estimated by Folin-Phenol reagent [16], gave a linear relationship with concentration over the 5–100 μg/mL range. HPLC analysis was performed to confirm spectrophotometric results. Residues were dissolved in an appropriate volume of mobile phase (1 mL for all samples, except for K8 control, 5 mL), filtered through a 0.45 μm disposable syringe filter (Micro Filtration Systems®) prior to injection into the chromatograph. Aliquots (20 μL) were injected on HPLC column and analysis were accomplished using a Shimadzu® Liquid Chromatograph, equipped with an LC-10AD pump, a Rheodine® injector, an SPD-10A UV detector, a CBM-101 Communications Bus Module, and a Class-CR10 Workstation system. A reverse-phase Shimpack® GLC-ODS (M) column (150 × 4.6 mm, 5 μm) was used, at room temperature, together with the same type of pre-column (10 × 4.6 mm). The mobile phase was acetonitrile-isopropanol-8.10^−2^ *M* phosphoric acid (35:10:55) with a flow rate of 0.8 mL/min for an isocratic run of 30 min. Absorbance of samples and standard was detected at 360 nm. Retention times and peak areas were calculated by Class-CR10 software. Comparison of sample retention times with that of the standard identified the presence of citrinin in the samples. The relationships between peak height and area and the amount injected were linear over the ranges 2.5–50 ng.

### 2.6. Recovery Experiments

Extraction procedure for spectrophotometric experiments was validated by the addition of citrinin standard (1mg/ml of ethanol: 10, 25 and 50 μL) to liquid PD (3 mL), extracted, spotted on plates and analyzed, as has been described for samples. Known aliquots of citrinin (5–50 μg) were also spotted, developed, detected, eluted, and estimated as samples to check on their recovery from plates. The percentage of added citrinin recovery in PD medium varied between 38% (for 10 μg) and 52% (for 50 μg), while recovery of known amounts of citrinin from plates averaged 98.4%.

### 2.7. Statistical Analysis

Statistical significance between control and experimental rates was calculated according to Turkey’s Test, using the Graph Pad Prisma program V.5 (Graph Pad Software).

## 3. Results and Discussion

**Figure 1.**
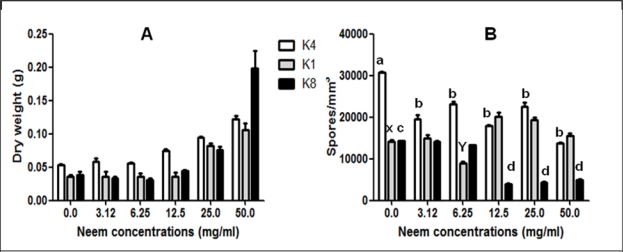
Effect of NLE added on PD medium on dry weight (A) and on spore numbers (B) of *P citrinum* (isolates K1, K4 and K8). Bars indicate standard deviation for experiments carried out in four replicates. Different small letters over the columns indicate statistically significant differences (*p<0.05*) for K4 spore numbers (a, b), K1 spore numbers (x, y) and K8 spore numbers (c, d).

Figure 1A shows the effects of different concentrations of NLE on *P. citrinum* isolates, grown on PD. Although fungus growth was maintained low at the initial concentration of NLE, fungal growth increased in neem concentrations up to 12.5 mg/mL.

Similar growth results were obtained by other authors, working with *P. citrinum* [18, 19]. The growth patterns were similar to growth of *P. expansum* [10] and consistent with the findings of other authors studying neem extracts in *Aspergillus*, in which the formation of toxins was prevented but not fungal growth [7–9]. The ability of the NLE to affect mycelium growth varied among isolates. Figure 1B shows no complete inhibition of spores amount after the incubation but, different patterns among isolates were observed: there is a significant decrease (p<0.05) on K4 spore numbers when 3.12 to 50 mg/mL of neem extract was added to medium and maintained for the next concentrations. There was a significant decrease (p<0.05) at 6.25 mg/mL of neem extract in isolate K1, however, an increase at 12.5 mg/mL and another decrease at 50 mg/mL occurred. Isolate K8 decreased significantly (p<0.05) at 12.5 to 50 mg/mL of neem extract (Values have been calculated by Turkey’s Test).

**Figure 2.**
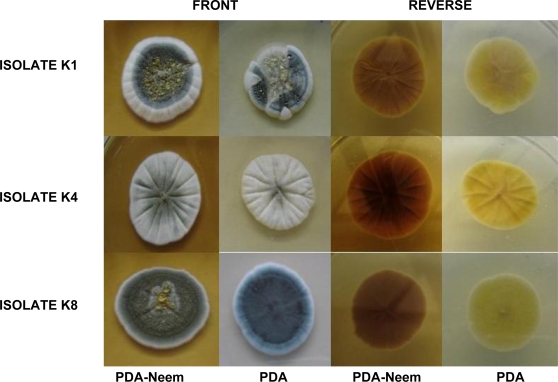
*P. citrinum i*solates (isolates K1, K4 and K8) grown on PDA and on PDA-NLE as described in ‘Materials and Methods’.

Direct contact of fungus with NLE on PDA surface resulted in differences between macroscopic colonies features but not at microscopic ones. Colonies grown on PDA-neem (Figure 2) were not only bigger in size than control colonies but their appearance differed too: colonies were radially sulcate, moderately deep and produced exudates.

The anterior part of colonies on PDA-neem was more intensely green with irregular margins. Microscopic observations revealed same sized conidia and conidiophore diameter; no alteration in asexual reproduction development, typical branching of conidiophores and normal conidiophore appearance; typical size and morphology of spores (results not shown). Comparisons were also made with the morphologic features of *P. citrinum* described in the literature [20].

**Figure 3.**
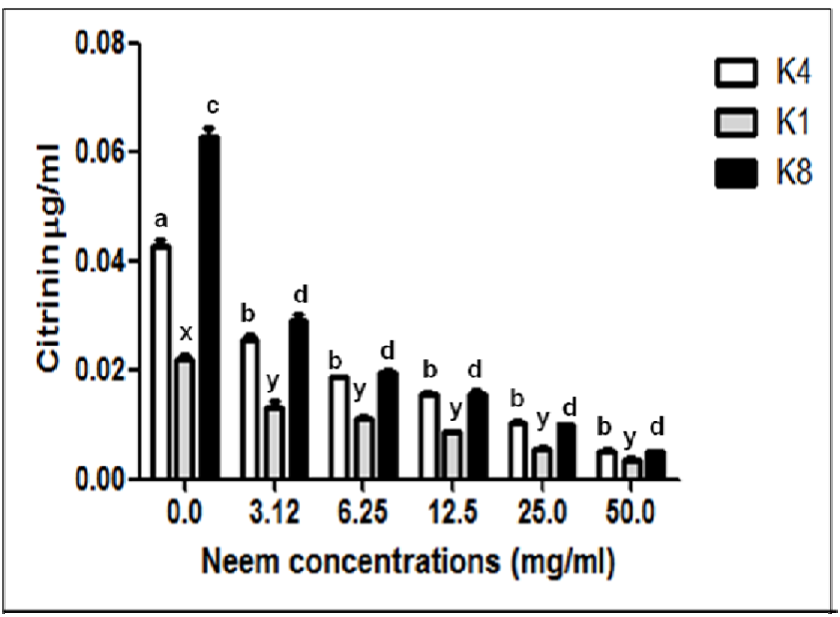
Citrinin production from *P. citrinum* isolates determined by spectrophotometric assay described in materials and methods. Bars indicate standard deviation for experiments carried out in four replicates. Different small letters over the columns indicate statistically significant differences (*p<0.05*) for isolate K4 (a, b), K1 (x, y) and K8 (c, d).

**Figure 4.**
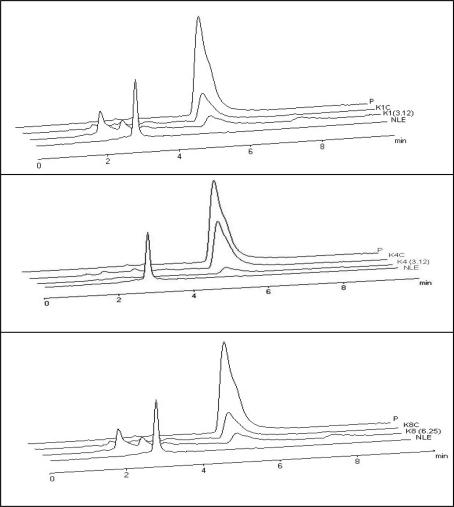
HPLC elution profiles from *P. citrinum* isolates, as described in ‘Materials and Methods’. **(P)** Citrinin standard; (**K1C**), (**K4C**), (**K8C**-dil.1/5) controls of citrinin production by *P. citrinum* isolates grown on PD without addition of neem extract; (**K1, K4)** Citrinin from *P. citrinum* isolates grown on PD with neem extracts (3.12 mg/mL) and (**K8)** Citrinin from *P. citrinum* isolates grown on PD with neem extracts (6.25 mg/mL); (NLE) neem leaf extract at 12.5 mg/mL.

Figure 3 shows the effects of NLE on citrinin production by *P. citrinum* isolates in PD broth, determinated by spectrophotometric experiments. After 21 days, quantitative determination [17] of the extracts from liquid culture media demonstrated inhibition of citrinin production by three isolates of *P. citrinum* on media with NLE. Inhibition of citrinin production does not appear to be simply a function of mycelia mass. The quantification of citrinin was also done by HPLC by comparing the retention times of the culture extracts with that of an authentic sample of citrinin. Standard and citrinin from samples were eluted between 5 to 6 min., as seen in Figure 4. The small peaks at 2–3 min. retention times (K1, 3.12 and K8, 6.25 mg/mL) were not identified and may be co-production metabolites of fungal growth.

Neem extracts reached 87.16% inhibition on K4 citrinin production at NLE 3.12 mg/mL, 85.86% inhibition on K1 citrinin production at NLE 3.12 mg/mL, and 94.86% inhibition on K8 citrinin production at NLE 6.25 mg/mL (Table 1). HPLC results confirmed spectrophotometer and TLC experiments and showed higher sensitivity. Generally, toxin production decreases as mycelium formation decreases. However citrinin inhibition was more significant than mycelial alteration.

Other authors [6–8, 21, 22] have also reported blocking of aflatoxin production with an apparently normal fungus growth and demonstrated that antifungal potentiality against growth may not coincide with the inhibitory potential of toxin production. Current analysis also revealed that although the NLE delayed the citrinin production, the three isolates regained their toxigenic capacity when re-cultivated without NLE (results not shown).

Current research offers the possibility of developing methods for controlling mycotoxin production by fungi coupled to novel environmentally safe agrochemicals of plant origin.

## 4. Conclusions

Neem is a millenary tree native to India where it has traditionally been used for centuries for treatment of human ailments and pest control. Studies on the various parts of the tree have increased significantly and its active principles have interesting potential for use in integrated pest management and alternative medicines. In current research, we have studied the potential of Neem leaf extract on inhibiting citrinin production by three isolates of *Penicillium citrinum* in culture. The fungi growth was not reduced, although there were differences between colony macroscopic characteristics in controls and treatments. There is also evidence given with these studies and in many others that fungi species react differently to compounds from the Neem tree. Additional research is needed to determine the potential usefulness of Neem products in fungi control programs.

## Figures and Tables

**Table 1.** Effect of NLE (mg/ml) on citrinin production (ng/ml) by *Penicillium citrinum* a (isolates K1, K4 and K8).

Isolate	b NLE (mg/ml)	Citrinin (μg)^c^ spectrophotometric assay	Reduction (%)	Citrinin (ng)^d^ HPLC analyses	Reduction (%)
K1	0.0	2.17 × 10^−2^	-	1.23 × 10^4^	-
	3.12	1.33 × 10^−2^	39.1%	0.175 × 10^4^	85.86%
	6.25	1.1 × 10^−2^	49.4%	-	
	12.5	0.8 × 10^−2^	60.9%	-	-
	25.0	0.5 × 10^−2^	74.7%	-	-
	50.0	0.3 × 10^−2^	83.9%	-	-
K4	0.0	4.25 × 10^−2^	-	3.28 × 10^4^	-
	3.12	2.55 × 10^−2^	40%	0.42 × 10^4^	87.16%
	6.25	1.80 × 10^−2^	56,5%	-	-
	12.5	1.55 × 10^−2^	63,5%	-	-
	25.0	1.03 × 10^−2^	75.9%	-	-
	50.0	0.5 × 10^−2^	88,2%	-	-
K8	0.0	6.3 × 10^−2^	-	10.7 × 10^4^	-
	3.12	3.0 × 10^−2^	53.7%	-	-
	6.25	2.0 × 10^−2^	68.6%	0.55 × 10^4^	94.86%
	12.5	1.6 × 10^−2^	75.0%	-	-
	25.0	1.0 × 10^−2^	84.0%	-	-
	50.0	0.5 × 10^−2^	92.0%	-	-

a Results are the means of two experiments with four replicates each, determined 21 days after incubation.

b NLE were prepared as described in ‘Material and Methods’.

c Values obtained by spectrophotometric assay are means of four replicates.

d Values obtained by HPLC analyses are means of four replicates.
